# Selection of red fluorescent protein for genetic labeling of mitochondria and intercellular transfer of viable mitochondria

**DOI:** 10.1038/s41598-022-24297-0

**Published:** 2022-11-18

**Authors:** Isamu Taiko, Chika Takano, Masayuki Nomoto, Shingo Hayashida, Kazunori Kanemaru, Toshio Miki

**Affiliations:** 1 Department of Physiology, Nihon University School of Medicine, Tokyo, Japan; 2 Division of Microbiology, Department of Pathology and Microbiology, Nihon University School of Medicine, Tokyo, Japan; 3 Department of Pediatrics and Child Health, Nihon University School of Medicine, Tokyo, Japan; 4 Division of Respiratory Medicine, Department of Internal Medicine, Nihon University School of Medicine, Tokyo, Japan

**Keywords:** Biological techniques, Imaging, Fluorescence imaging

## Abstract

The phenomenon of intercellular mitochondrial transfer has attracted great attention in various fields of research, including stem cell biology. Elucidating the mechanism of mitochondrial transfer from healthy stem cells to cells with mitochondrial dysfunction may lead to the development of novel stem cell therapies to treat mitochondrial diseases, among other advances. To visually evaluate and analyze the mitochondrial transfer process, dual fluorescent labeling systems are often used to distinguish the mitochondria of donor and recipient cells. Although enhanced green fluorescent protein (EGFP) has been well-characterized for labeling mitochondria, other colors of fluorescent protein have been less extensively evaluated in the context of mitochondrial transfer. Here, we generated different lentiviral vectors with mitochondria-targeted red fluorescent proteins (RFPs), including DsRed, mCherry (both from *Discosoma* sp.) Kusabira orange (mKOκ, from *Verrillofungia concinna*), and TurboRFP (from *Entacmaea quadricolor*). Among these proteins, mitochondria-targeted DsRed and its variant mCherry often generated bright aggregates in the lysosome while other proteins did not. We further validated that TurboRFP-labeled mitochondria were successfully transferred from amniotic epithelial cells, one of the candidates for donor stem cells, to mitochondria-damaged recipient cells without losing the membrane potential. Our study provides new insight into the genetic labeling of mitochondria with red fluorescent proteins, which may be utilized to analyze the mechanism of intercellular mitochondrial transfer.

## Introduction

Mitochondria are ATP-producing organelles responsible for cellular energetics and metabolism. Mitochondrial diseases cause a primary defect in oxidative phosphorylation and can involve any organ or tissue. The clinical features are heterogeneous, which could delay diagnosis and treatment. Moreover, the treatment of patients remains a challenge, although several therapeutic options are being investigated^1^.

Recent studies revealed that mitochondria are actively transported from healthy donor cells to cells with damaged mitochondria^2,3^. This phenomenon is called intercellular mitochondrial transfer and may be one of the innate survival systems of eukaryotic cells. It is hypothesized that this phenomenon can be applied to treat diseases associated with mitochondrial damage. Intercellular mitochondrial transfer from and between tissue stem cells has been reported to have therapeutic potential^4^. Thus, efforts to elucidate the mechanism of mitochondrial transfer have attracted great attention in the field of stem cell biology.

Human amniotic epithelial cells (hAECs) are a type of placental stem cell that possess pluripotent stem cell-like differentiation potential, immunomodulatory, and anti-inflammatory properties and are therefore considered to have prospects for cell-based therapy^5,6^. Since the placenta is the youngest donor tissue, placental mitochondria are considered to be less damaged by aging and environmental factors. Thus, hAECs could be ideal donor stem cells for therapeutic mitochondrial transfer. To conduct further investigations of mitochondrial transfer from hAECs, reliable tools for mitochondrial visualization are desired.

Imaging fluorescently labeled mitochondria with small molecules such as MitoTracker is a promising technique that can visualize mitochondrial transfer, thereby contributing to an understanding of the mechanisms underlying this process. However, small molecules often leak from labeled organelles, which can lead to false-positive results^7^. Moreover, the covalent labeling of mitochondrial matrix proteins with fluorescent molecules is sometimes harmful to mitochondrial respiration^8,9^. These problems can be addressed by genetically expressing fluorescent proteins with a mitochondrial targeting signal (MTS)^10^. Genetically encoded fluorescent tags enable the long-term observation of mitochondria even in vivo^11^. Enhanced green fluorescent protein (EGFP) is the most widely used fluorescent protein tag for studying various cellular functions, including labeling mitochondria. In addition to EGFP, distinct color variations of fluorescent proteins are required to study mitochondrial transfer between two different cells.

Red fluorescent protein is the second most widely used fluorescent protein tag for labeling organelles in living cells. DsRed and its variants are often used for labeling mitochondria in studying mitochondrial transfer^12–14^. However, DsRed and some other red fluorescent proteins can oligomerize, which can affect protein folding and translocation to the mitochondria, causing mislocalization of the proteins^15^. The mislocalization of fluorescent proteins may be responsible for failure to detect mitochondrial transfer, especially by flow cytometric analysis or fluorescence-activated cell sorting. Thus, appropriate red fluorescent proteins are essential for the precise detection of mitochondrial transfer.

In this study, we generated four lentiviral vectors with different red fluorescent proteins targeted to mitochondria by MTS and assessed the intracellular fluorescence localizations in hAECs. Despite these vectors sharing the same backbone sequence, including promoter and MTS sequences, DsRed and its derivative mCherry formed punctate aggregates, which localized in lysosomes. Furthermore, we successfully detected mitochondrial transfer by using TurboRFP.

## Results

### Selection of mitochondria-targeted fluorescent proteins and design of lentiviral vector constructs

**Figure 1:**
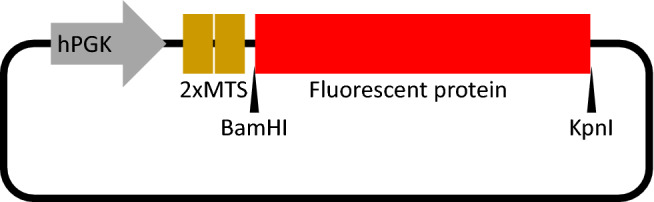
Scheme of a mitochondria-targeted fluorescent protein expression vector. The selected fluorescent protein genes were inserted between BamHI and KpnI restriction enzyme sites of pLKO-based backbone, each preceded by a human phosphoglycerate kinase (hPGK) promoter and a tandem mitochondrial targeting signal (MTS) of the human COX8A gene.

To evaluate different red fluorescent proteins, the following four commonly used red fluorescent proteins derived from different organisms were selected; DsRed derived from coral *Discosoma* sp. and its monomeric variant mCherry^16,17^, TurboRFP derived from the sea anemone *Entacmaea quadricolor*, and the monomeric orange-shifted fluorescent protein mKOκ derived from the stony coral *Verrillofungia concinna*^18,19^ (Table 1). DNA sequences encoding these red fluorescent proteins were inserted into a common lentiviral vector (pLKO) backbone (Fig. 1). We also generated the same backbone lentiviral vector with the EGFP gene for comparison. To prevent extrinsic protein overloading in mitochondria, we employed a human phosphoglycerate kinase (hPGK) promoter for a moderate level of gene expression^20,21^. Two human COX8A gene sequences were used as a tandem MTS to prevent the leakage of fluorescent protein into the cytosol^22^.

**Table 1:** Reported properties of fluorescent proteins.

Protein	Oligomerization	Origin	Excitation maximum (nm)	Emission maximum (nm)	Extinction coefficient (M^−1^ cm^−1^)	Quantum yield	p*K*a	References
EGFP	Weak dimer	*Aequorea victoria*	488	507	55,900	0.6	6.0	^31,32^
DsRed	Tetramer	*Discosoma* sp	558	583	57,000	0.79	4.7	^13,14^
mCherry	Monomer	*Discosoma* sp	587	610	72,000	0.22	4.5	^15^
TurboRFP	Dimer	*Entacmaea quadricolor*	553	574	92,000	0.67	4.4	^16,31^
mKOκ	Monomer	*Verrillofungia concinna*	551	563	105,000	0.61	4.2	^17^

### Differential intracellular impact of the mitochondria-targeted fluorescent proteins

**Figure 2:**
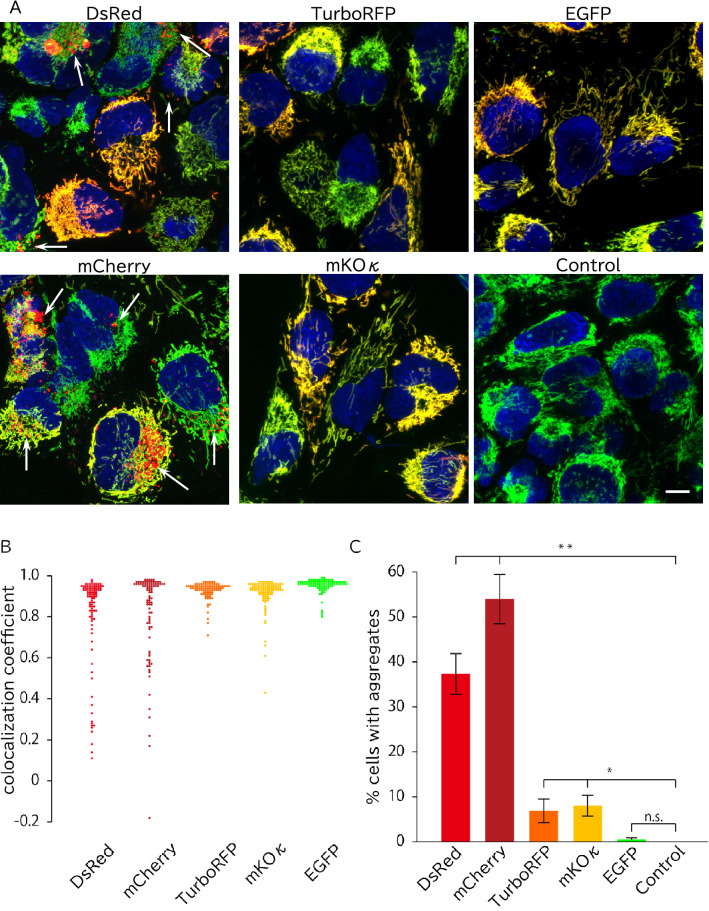
Aggregate formation of mitochondria targeted mCherry and DsRed proteins. (**A**) Representative images of iAECs transduced with each mitochondria-targeted protein or uninfected control stained with MitoTracker and Hoechst. Merged images of each color are shown. Blue: Hoechst 33342. For DsRed, mCherry, TurboRFP mKOκ and uninfected control, green: MitoTracker Green, red: each fluorescent protein. For EGFP, green: EGFP, red: MitoTracker Orange. Scale bar = 10 µm. (**B**) Distribution of the colocalization coefficients of fluorescent proteins and MitoTracker of the cells. Each group included at least 80 cells from 7 optical fields. (**C**) Proportion of cells with aggregates in the mitochondria-targeted fluorescent protein-transduced cell population. Data are shown as the mean ± SD, n = 3 biological replicates. Each replicate included at least 80 cells from 7 optical fields. **p < 0.01, *p < 0.05 n.s.: not significant (p > 0.05); Tukey's HSD test. All cells were infected with lentivirus at an MOI of 5.

**Figure 3:**
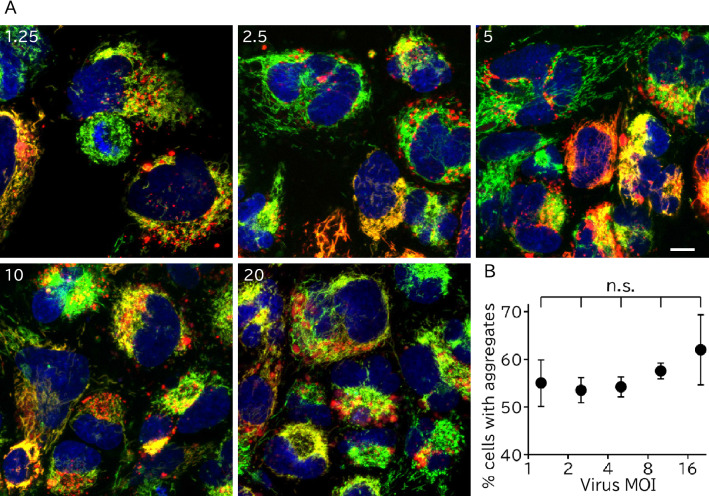
Aggregate formation is not dependent on the virus dose. (**A**) Representative images of iAECs transduced with serially diluted lentivirus containing mitochondria-targeted mCherry (red) stained with MitoTracker Green (green) and Hoechst 33342 (blue). The top-left numbers in the images indicate the virus MOI. Scale bar = 10 µm. (**B**) The proportion of cells with aggregates is plotted against the virus titer. Data are shown as the mean ± SD, n = 3 biological replicates. Each replicate included at least 80 cells from 7 visual fields. n.s.: not significant (p > 0.05); Tukey's HSD test.

**Figure 4:**
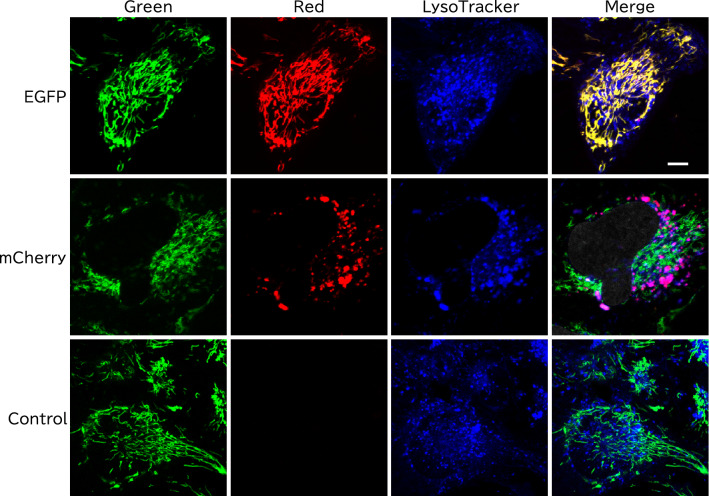
Colocalization of mCherry aggregates with lysosomes. Representative images of iAECs transduced with mitochondria targeting mCherry or EGFP or uninfected controls stained with MitoTracker Green (green, for mCherry and control) or Orange (red, for EGFP) and LysoTracker Deep Red (blue). Scale bar = 10 µm.

Lentivirally transduced fluorescent proteins had no notable cytosolic leakage and localized to mitochondria in immortalized hAECs (iAECs) (Fig. 2). We examined the precise subcellular localization of the mitochondria-targeted proteins by costaining with MitoTracker dye (Fig. 2A,B). Mitochondria-targeted EGFP, TurboRFP, and mKOκ were costained with MitoTracker dye and did not form visible aggregates or cause abnormal mitochondrial structure in most cells. In contrast, mitochondria-targeted mCherry and DsRed formed MitoTracker-unstained granular aggregates around the nucleus in 37 ± 4.5% and 54 ± 5.5% of cells, respectively (Fig. 2C). These aggregates had higher fluorescence intensity than mitochondria-localized fluorescent proteins. These cells also had normal mitochondrial morphology. In some of the cells, both aggregated and accurately mitochondria-targeted mCherry and DsRed coexisted. In other cells, only aggregates were present. These aggregates emerged even at the low viral dose and were not significantly changed in a viral dose-dependent manner (Fig. 3). To investigate whether the mCherry aggregates were present in the lysosome, lysosomes were visualized with LysoTracker. As expected, most of the aggregated mCherry (98.3% in 38 cells) was costained with LysoTracker, while mitochondria-localized mCherry was completely distinct (Fig. 4).

### Toxicity and photostability of red fluorescent proteins

**Figure 5:**
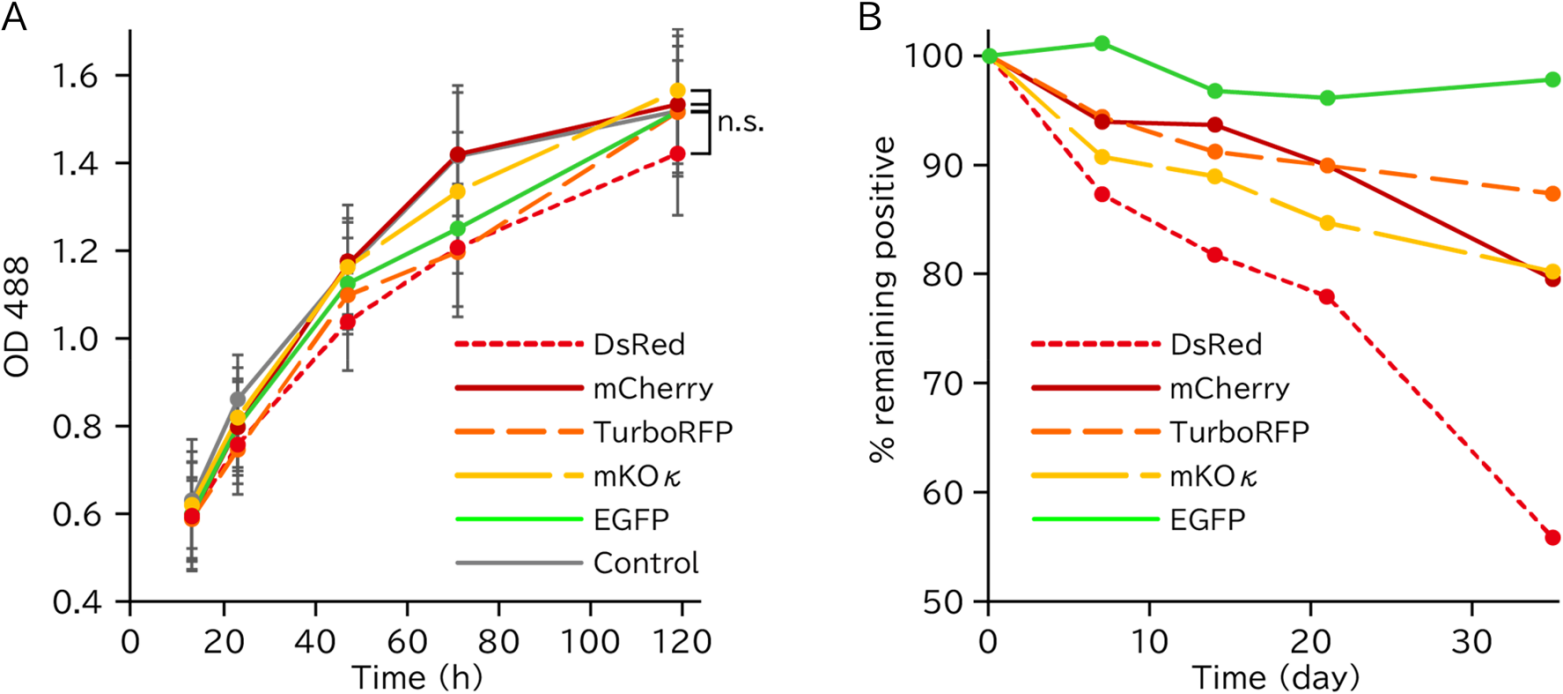
Cytotoxicity of mitochondria-targeted proteins. (**A**) Growth curve of iAECs transduced with the mitochondria-targeted protein or uninfected control. Data are shown as the mean ± SD, n = 3, n.s.: not significant (p > 0.05); Tukey's HSD test. (**B**) Time course of depletion of fluorescent positive cells analyzed by flow cytometry.

We next examined the toxicity of these red fluorescent proteins when expressed in mitochondria. We assessed the viability of cells stably transduced with mitochondria-targeted red fluorescent protein. Unexpectedly, the cell growth rates were almost comparable to each other and even to the control cells (Fig. 5A). All fluorescent proteins were maintained for one month. The fluorescence intensity and retained cell rate gradually decreased, except for that of EGFP, which exhibited no noticeable decrease even after one month (Fig. 5B).

**Figure 6:**
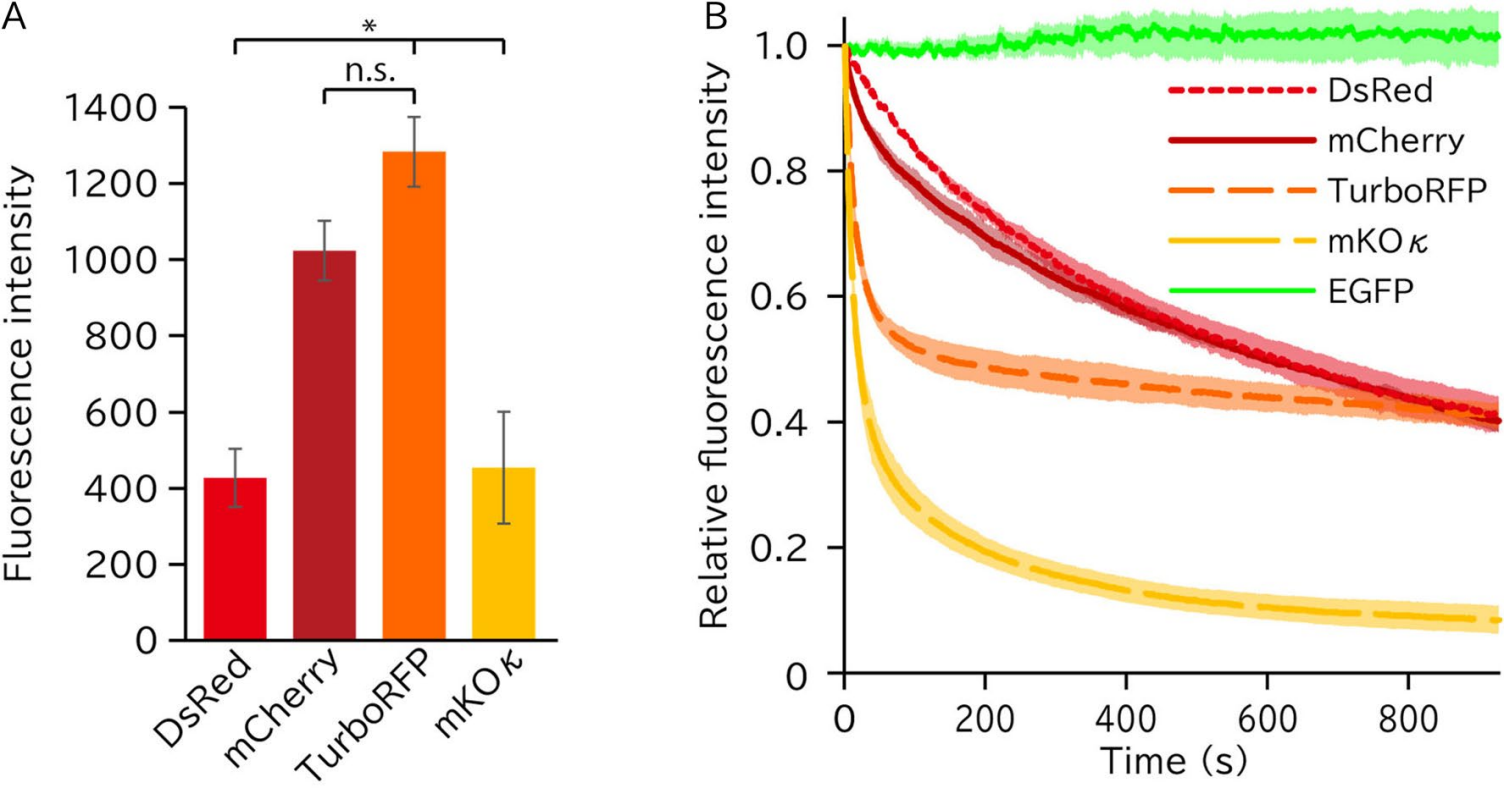
Brightness and photostability of the mitochondria-targeted proteins. (**A**) Average fluorescence intensity of mitochondria-targeted fluorescent proteins. n = 3 biological replicates. Each replicate included at least 80 cells from 7 visual fields. n.s.: not significant (p > 0.05); Tukey's HSD test. (**B**) Photobleaching of fluorescent proteins in iAECs during confocal imaging with a 488 nm (for EGFP) or a 555 nm (for other fluorescent proteins) excitation laser. The data are the average of at least three cells. Shaded areas indicate ± SD.

Since studies of mitochondrial transfer may require long-term observation of the transferred mitochondrion, the labeling fluorescent protein should possess sufficient brightness and photostability. We compared the fluorescence intensity and photobleaching profiles of these red fluorescent proteins in mitochondria. Among the four tested red fluorescent proteins, TurboRFP showed the highest fluorescence intensity (12,831 ± 917 A.U.) under the optical setups widely used to observe RFP (Fig. 6A). The fluorescent signal of TurboRFP was still sufficiently detectable even after paraformaldehyde fixation (Supplementary Fig. S1). The photostability was calculated from the change in fluorescent intensity after exposure. Although the fluorescence intensity of TurboRFP decreased quickly, it maintained approximately 50% intensity for up to 900 s of exposure (Fig. 6B).

In summary, we concluded that TurboRFP was the most suitable red fluorescent protein for exploring mitochondrial transfer.

### Intracellular transfer of viable TurboRFP-labeled mitochondria

**Figure 7:**
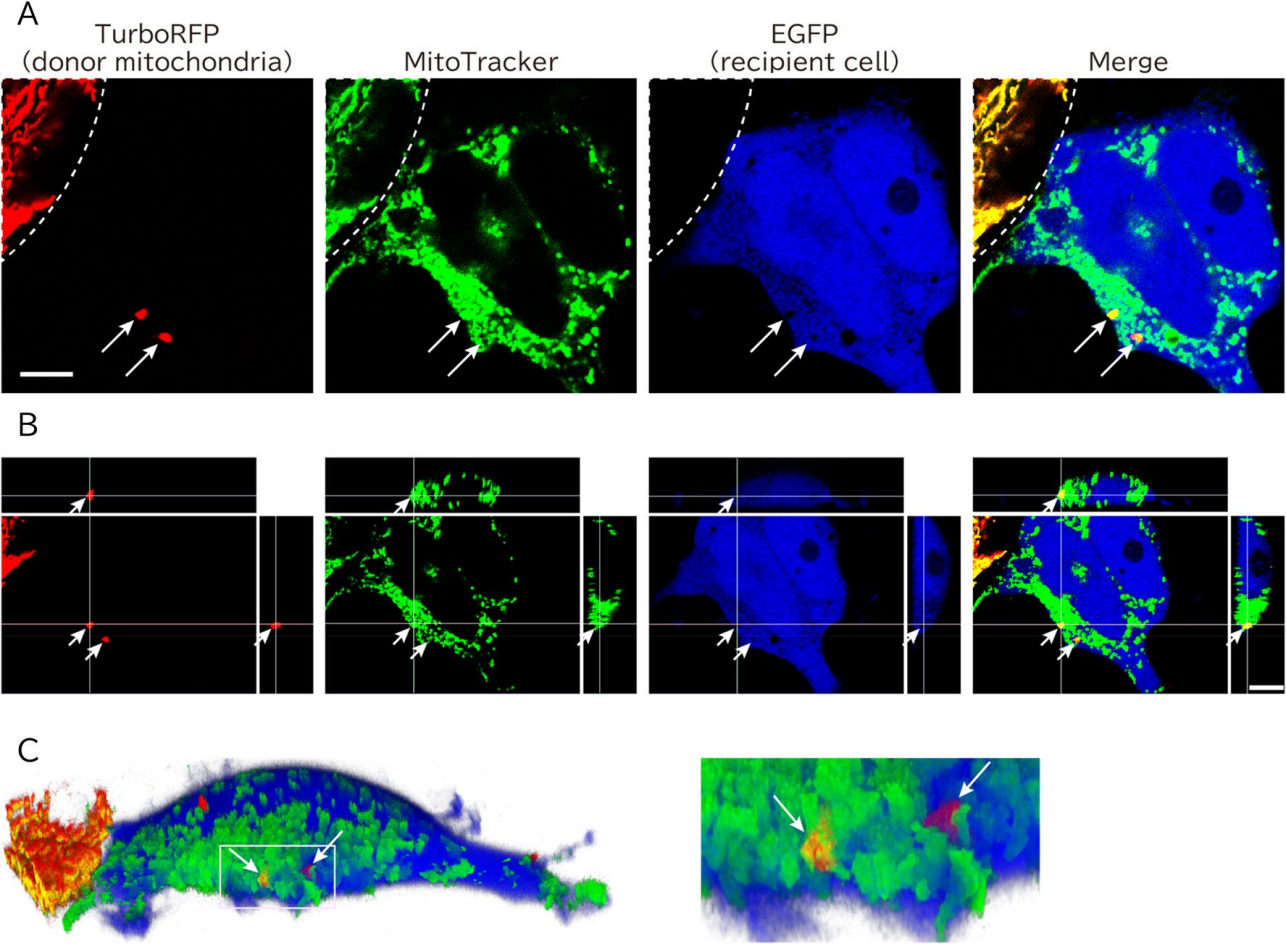
Mitochondria transfer from iAECs to damaged cells. (**A**) Representative images of transferred mitochondria. Stacked confocal microscopy images are shown. White arrows indicate transferred mitochondria. The white dotted line indicates a boundary of cocultured donor cell. (**B**) 3D reconstruction of z-slices of the corresponding regions shown in (**A**). Middle: One of the z-slice images shown in (**A**). Upper: An optical section of the corresponding region of the vertical bar shown in the middle panel. Right: An optical section of the corresponding region of the horizontal bar shown in the middle panel. Both horizontal and vertical bars shown in the upper or right panel indicate sectioned positions of the middle. (**C**) The 3D reconstructed image shown in (**B**). Left: Full view, right: an enlarged image of the rectangle at the left. White arrows indicate transferred mitochondria, as shown in (**A**). Note that TurboRFP-labeled mitochondria were inside the recipient cell and were stained by MitoTracker Deep Red, whose accumulation is dependent on mitochondrial membrane potential. Scale bar = 10 µm.

We tested whether mitochondrial transfer can be detected by labeling mitochondria with TurboRFP. As described above, hAECs should have the potential to transfer intact mitochondria into cells with damaged mitochondria. We utilized mitochondria-targeted TurboRFP in iAECs to label donor mitochondria. HEK293T cells were employed as recipient cells. We transduced HEK293T cells with cytosolic EGFP to distinguish donor iAECs. We adopted a hydrogen peroxide (H_2_O_2_)-induced damage model for mitochondrial dysfunction. Because mitochondrial DNA is more susceptible than nuclear DNA to H_2_O_2_, the short-term treatment of cells with an appropriate concentration of H_2_O_2_ can selectively damage mitochondrial DNA and thus induce mitochondrial dysfunction. Donor HEK293T cells were exposed to H_2_O_2_ for 60 min prior to the experiments. Damaged HEK293T cells were cocultured with iAECs expressing mitochondria-targeted TurboRFP for 20 h. Then, these cells were analyzed by confocal microscopy to properly distinguish the transferred intracellular red mitochondria from those overlaid on EGFP-positive cells. We found that TurboRFP-labeled mitochondria were transferred into EGFP-labeled HEK293T cells (Fig. 7). To evaluate the membrane potential of the transferred TurboRFP-positive mitochondria, the samples were further stained with MitoTracker Deep Red. 3D-reconstructed confocal microscopy imaging revealed that the TurboRFP-positive mitochondria in the EGFP-positive cells were costained with MitoTracker Deep Red, which indicated that the transferred mitochondria maintained the active membrane potential (Fig. 7C).

## Discussion

The results of this study showed that lentivirally transduced mitochondria-targeted DsRed and mCherry proteins formed a large number of bright punctate aggregates that were not localized in mitochondria. Aggregate formation did not affect the cell viability in this study, and thus the experiment could be carried out without noticing aggregation, which could lead to false conclusions. We further showed that these aggregates were localized mainly in lysosomes. Lysosomes are known to be able to release their contents by exocytosis machinery. The extracellularly released DsRed and mCherry aggregates could be internalized by other cells, including donor cells, through endocytosis or phagocytosis. These internalized aggregates may be mistaken for transferred mitochondria. This is an especially critical issue in the quantitative evaluation of intercellular mitochondrial transfer by fluorescence-activated flow cytometric analysis, which cannot easily distinguish internalized aggregates from transferred mitochondria based on their shape. Thus, although DsRed and mCherry are currently used in most mitochondrial transfer experiments, interpretation of the data requires care. This finding provides a first warning of the need to select appropriate red fluorescent protein for each experimental design.

We do not have compelling evidence to explain the mechanisms underlying the aggregate formation of MTS-fused DsRed and mCherry proteins in lysosomes. One possible reason is that the MTS or the linker peptide used in our experiments might affect protein folding, maturation, or localization. However, other proteins used here and in other experiments with this MTS and linker were precisely localized in the mitochondria^23^. Alternatively, excessive expression of the proteins might induce aggregate formation in lysosomes. However, we showed that aggregates appeared even with a low multiplicity of infection (MOI) and did not increase with the viral dose. Furthermore, since the hPGK promoter is a weak promoter in most mammalian cell types, the proteins were unlikely to be expressed in excessive amounts. Katayama et al. reported that cytoplasmic DsRed proteins can accumulate in lysosomes, possibly by autophagocytosis^24^. Costantini et al. also reported that Golgi complex targeting sequence fused mCherry proteins accumulated in lysosomes^25^. Thus, lysosomal aggregation of mitochondrial-targeted DsRed and mCherry could be induced by autophagocytosis through their inherent feature because of their origin.

Our comparison study indicated that TurboRFP is suitable as an alternative to DsRed and mCherry for mitochondrial transfer experiments in AECs. TurboRFP is a bright and rapidly maturing red fluorescent protein and is also widely used. TurboRFP has appropriate excitation and emission wavelengths that overlap minimally with green fluorescent molecules such as EGFP or FITC and deep red fluorescent molecules such as Cy5 dye and is thus ideal for multicolor labeling strategies^18^. We showed that TurboRFP possesses brightness and photostability comparable to those of mCherry and exhibits no notable toxicity. Most importantly, we could detect mitochondrial transfer by labeling mitochondria with TurboRFP. The investigation of dynamics and morphology is essential for mitochondrial research. Our comparison study of mitochondrial labeling is also informative for other mitochondrial experiments.

In this study, we showed for the first time that hAECs can donate their mitochondria to H_2_O_2_-damaged cells. The ability of mitochondria to transfer into damaged cells has been reported in various cell types, including MSCs^4^. However, until now, there has been no report that hAECs can transfer mitochondria. As a donor of mitochondria transfer, hAECs have certain advantages associated with their unique characteristics^5^. First, hAECs contain healthy, virtually undamaged mitochondria. Since hAECs are placenta-derived neonatal cells, they have minimal exposure to aging and environmental damage. Second, large quantities of hAECs can be easily and inexpensively isolated from a placenta with minimum ethical concerns^26^. The placenta is usually discarded as clinical waste after delivery. Thus, no additional invasive procedures are required to obtain hAECs. Third, hAECs can be safely transplanted into other individuals. Due to their genomic stability, hAECs lack tumorigenicity and do not form any type of tumor upon transplantation^27,28^. Moreover, hAECs have immunomodulatory properties, which can benefit the control of immune-mediated inflammation and rejection after cell transplantation^29^. Taken together, hAECs can be considered one of the best donor cells for mitochondrial transfer. Our study paves the way for the cell-based therapeutic application of hAECs to treat damaged mitochondria.

In summary, we characterized various red fluorescent proteins for mitochondrial labeling. Among them, mCherry and DsRed proteins tended to accumulate in the lysosome. Moreover, we showed that hAECs whose mitochondria were labeled with TurboRFP possessed the ability to transfer functional mitochondria.

## Methods

### Cell culture

HEK293T cells were purchased from GeneHunter and cultured on collagen-coated dishes (Nunc) in DMEM (Gibco) supplemented with 10% fetal bovine serum (Gibco), 100 U/ml penicillin and streptomycin, and 2.5 ng/ml amphotericin B (Gibco).

Previously established immortalized hAEC line cells were used^30^. Briefly, primary human amniotic epithelial cells from five different donors were immortalized by using an SV40 lentiviral vector (pLenti-SV40-T + t, Applied Biological Materials Inc., Richmond, BC Canada). The established immortalized hAEC lines were morphologically evaluated, and one line (iAE129) was selected and used for further studies.

iAECs were cultured in DMEM supplemented with 10% fetal bovine serum (Nichirei), 200 μM l-glutamine (Gibco), 1 × nonessential amino acids (Gibco), 5.5 µM 2-mercaptoethanol (Gibco), 5 ng/ml EGF (PeproTech), 100 U/ml penicillin and streptomycin, and 2.5 ng/ml amphotericin B.

All cells were cultured in 5% CO_2_ at 37 °C.

### Lentiviral vector production

Lentiviral vectors (transfer plasmids) were constructed by cloning each fluorescent gene into a backbone of a common second generation lentiviral vector plasmid with hPGK promoter (pLKO). Two human COXA8 genes were inserted under the promoter as a tandem MTS (Fig. 1).

For lentiviral particle production, HEK293T cells were seeded on collagen-coated 10 cm dishes at 70–80% confluency. Cells were cotransfected with 4 μg of lentiviral vector plasmid along with 4 μg psPAX2 and 2 μg of pMD2. G (packaging plasmids, Addgene plasmids 12,260 and 12,259). Transfection mixtures were prepared in 500 μL of Opti-MEM I (Life Technologies) containing the DNA and 27 μL of Lipofectamine 3000 (Life Technologies) according to the manufacturer's protocol. The medium was changed 12 h after transfection, and lentiviral supernatants were collected after 24 and 48 h. Collected lentiviral supernatants were concentrated via ultracentrifugation, and the medium was exchanged with phosphate buffered saline.

### Imaging

To image mitochondria-targeted proteins, iAECs were seeded on a 24-well plate (Nunc) at a density of 50,000 cells/well and infected with lentivirus after 12 h. Cells were seeded on glass bottom dishes (Matsunami) 2 days before imaging. Cells were loaded with 100 nM MitoTracker green or Orange CMTMRos (Life Technologies) and 500 ng/ml Hoechst 33342 for 30 min in culture medium at 37 °C. For lysosome staining, cells were loaded with 100 nM LysoTracker Deep Red (Life Technologies) in addition to the above. Then, the cells were washed twice with medium and imaged in 5% CO_2_ at 37 °C in the culture medium.

For the mitochondrial transfer experiment, HEK293T cells transduced with cytosolic EGFP were seeded on collagen- and poly-l-lysine-coated glass-bottom dishes for two days. The cells were treated with 300 μM hydrogen peroxide for 60 min at 37 °C and then cocultured with iAECs transduced with mitochondria-targeted TurboRFP in fresh iAECs medium. After 20 h of culture, the cells were loaded with MitoTracker Deep Red (Life Technologies) and imaged at room temperature. We conducted mitochondrial transfer experiments for 3 times and detected mitochondrial transfer in all experiments.

All fluorescence images were captured using a confocal microscope (TCS SP8, Leica) equipped with a 63 × objective (NA 1.40, HC PL APO, Leica) with excitation/emission wavelengths (nm) of 405/415–455 for Hoechst 33342, 488/496–543 for MitoTracker Green and EGFP, 555/565–620 for MitoTracker Red and the red fluorescent proteins, and 638/570–700 for LysoTracker Deep Red and MitoTracker Deep Red.

3D reconstructed images were obtained by Leica LAS X software (Version 3.5.5) and other images were analyzed by imageJ software (Version 1.52p).

### Cell growth and flow cytometry experiments

iAECs were seeded on a 24-well plate at a density of 50,000 cells/well and infected with lentivirus at an MOI of 5. After 4 days of infection, the cells were passaged and cultured for 6 days to exclude virus toxicity. Then, the cells were reseeded on 96-well plates (Nunc) at a density of 5000 cells/well. Cell proliferation was measured 12, 24, 48, 72, and 120 h after seeding by using a Cell Counting Kit-8 (Dojindo) according to the manufacturer's protocol.

For flow cytometry analysis, cells were analyzed by a FACS Aria (BD) equipped with 488 (for EGFP) or 561 (for DsRed, mCherry, TurboRFP and mKOκ) nm laser and 530/30 (for EGFP) or 582/15 (for DsRed, mCherry, TurboRFP and mKOκ) nm emission filter. Cells were analyzed 6, 14, 21, 28 and 42 days after infection. Cells were passaged at intervals of 1 week.

## Supplementary Information

Supplementary Legends.

Supplementary Figures.

## Data Availability

The datasets used and/or analyzed during the current study are available from the corresponding author on reasonable request.
