# Supplementary material for: Selection of red fluorescent protein for genetic labeling of mitochondria and intercellular transfer of viable mitochondria
Source: Sci Rep. 2022 Nov 18;12:19841. doi: 10.1038/s41598-022-24297-0 (PMC9674635; doi:10.1038/s41598-022-24297-0)
Supplement: Supplementary file 1 — Supplementary Legends. [file 41598_2022_24297_MOESM1_ESM.docx]

Selection of red fluorescent protein for genetic labeling of mitochondria and intercellular transfer of viable mitochondria

**Authors**

Isamu Taiko^1^, Chika Takano^2,3^, Masayuki Nomoto^4^, Shingo Hayashida^3^, Kazunori Kanemaru^1^ and Toshio Miki^1^*

1: Department of Physiology, Nihon University School of Medicine, Tokyo, Japan

2: Division of Microbiology, Department of Pathology and Microbiology, Nihon University School of Medicine, Tokyo, Japan.

3: Department of Pediatrics and Child Health, Nihon University School of Medicine, Tokyo, Japan.

4: Division of Respiratory Medicine, Department of Internal Medicine, Nihon University School of Medicine, Tokyo, Japan

*: Correspondence should be addressed to Toshio Miki

E-mail address: miki.toshio@nihon-u.ac.jp

Supplementary Figure1

A representative image of iAECs transduced with mitochondria targeted TurboRFP after paraformaldehyde fixation. Blue: Hoechst 33342, red: TurboRFP. Scale bar = 10 µm.
