# Supplementary figures and images for: Selection of red fluorescent protein for genetic labeling of mitochondria and intercellular transfer of viable mitochondria
Source: Sci Rep. 2022 Nov 18;12:19841. doi: 10.1038/s41598-022-24297-0 (PMC9674635; doi:10.1038/s41598-022-24297-0)

Supplementary Figure1

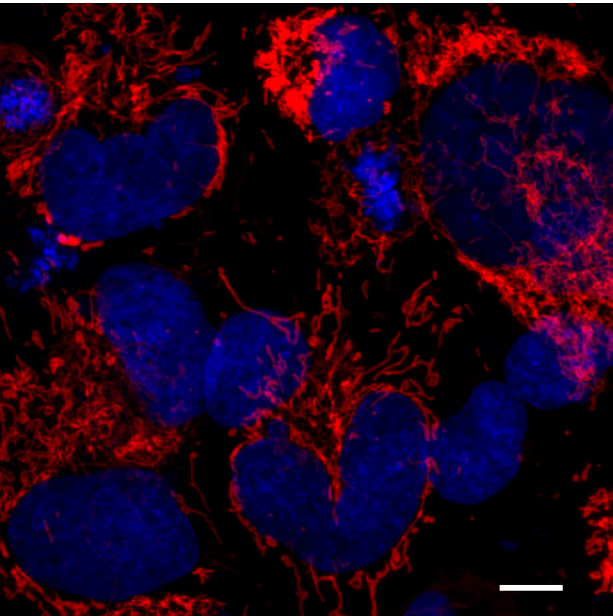

Supplement: Supplementary file 2 — Supplementary Figures. [file 41598_2022_24297_MOESM2_ESM.pdf]
